# An Account of Some of the Most Important Diseases Peculiar to Women

**Published:** 1832

**Authors:** 


### REVIEWS

### AND BIBLIOGRAPHICAL NOTICES.

---

Art. V.—*An account of some of the most Important Diseases peculiar to Women.* By Robert Gooch, M. D. From the Second London edition. Philadelphia: E. L. Carey and A. Hart—Chesnut street, 1832.

This work which is now, for the first time, presented to the Profession in the United States, comes to them with high claims to their notice. The opportunities of the author for observation have been extensive, he having occupied the post, for many years, of physician to the Lying-in Hospitals of Westminster, and of Lecturer on Midwifery at St. Bartholomew's, besides being extensively employed in private practice.

The principal design of this work is to give a full and particular account of certain forms of disease which are generally appropriated to the obstetric practitioner, that have not received the attention they deserve from writers upon that subject. It is a fact of general notoriety, that monographs upon this part of the profession are more rarely met with than upon other branches of Medical Science. If there is any thing in the importance of the subject, or in the embarrassing circumstances under which the young practitioner is frequently called upon to make his decision, we are not aware of it. General systems of medicine are, of necessity, inaccurate and defective, and the physician who is familiar only with compendiums, is very often thrown upon his own resources, under circumstances of difficulty and embarrassment that must subject the life of his patient to very great hazard.

To supply this defect in some degree is the object of the present work. Before proceeding to a review of it, we shall take the liberty of transcribing the author's advice to the young Practitioner of Midwifery. "Your task," says he, speaking to the young practitioner, "will go on prosperously, the sooner you have ceased to read, and begun to observe and think. Do not, however, attempt to dispense with reading, but dispatch it as speedily as is consistent with accuracy. Keep a note book, read the most esteemed original writers on the most important subjects of your art, and while reading them, briefly note down those points you wish to remember, so as to have no occasion ever to look into the book again. Provided you get the points of the work, the more briefly you do it the better; if you are skilful at this, you will find a page will hold a pamphlet, and that twenty pages will hold a bulky volume. Thus your manuscript volume will become a Bibliotheca of your branch of medicine, and you will never afterwards have occasion to consult the books themselves."

After having pursued this course until he is acquainted with the general principles of the profession, he advises him to watch cases attentively, and take notes of their important particulars—"a short description of the leading circumstances, with an equally short mention of the reflections which they suggest." Knowledge thus acquired, impressed and illustrated, by the study of morbid anatomy, conducted upon similar principles, will render a man, in a few years, competent to advise and act in the most difficult emergencies.

There are some writers, the author well remarks, whom it would be wrong to abandon thus; "master minds, whom we return to again and again, not so much for the knowledge they contain, but to observe how their minds worked; and the older we grow the fonder we become of them; such in England are Harvey in Physiology and Sydenham in Medicine.

It is unnecessary, we presume, to enforce upon our juvenile readers this mode of study. More of us are acquainted with its importance than have sufficient perseverance to carry it into execution. If generally adopted, a higher standard of medical education and of medical acquirements would at once be established. Physicians generally would not only be better qualified to practice and to write, but to distinguish the substance from the tinsel in the writings of others, and be prepared to demand from those whose office it is to instruct the profession either by writing or by lectures, superior qualifications than have generally fallen to the lot of those who occupy those important stations in this, or any other country.

The first chapter of this work is devoted to the Peritoneal Fevers of Lying-in Women. The following is Dr. G's description of the essential symptoms of the disease.

> "Its essential symptoms are pain and tenderness over the abdomen, with a rapid pulse. It begins a few days after delivery, with pain of the abdomen, shivering succeeded by heat, and a quick pulse. As the disease advances, the milk becomes suppressed, the belly tumid, and the breathing short; when it terminates fatally, it does so commonly about the fifth day, but often in less than half that time, On opening the abdomen, the morbid appearences are not uniform, but the most common and remarkable are copious effusions of lymph and serum on the surface and in the cavity of the peritoneum."

Dr. G. refers to several remarkable peculiarities of this disease; 1. It is much more prevalent at some seasons than others. In populous cities, some cases, few in number, mild in their symptoms, and comparatively manageable, are generally to be met with. At others, it prevails extensively as an epidemic, and with great fatality. 2. Another remarkable circumstance is, that when most prevalent, it is most fatal also. When it has been raging for some time, as the cases become less frequent, they become less fatal also, not so much on account of the increased skill of the physician, as the comparative mildness of the disorder. 3. It is not uncommon for the greater number of cases, when the disease is epidemic, to occur in the practice of one individual. Of this peculiarity Dr. G. relates several instances, and hence, as he remarks, the suspicion has very naturally originated, that the disease might be communicated in the clothes of the practitioner, or the furniture of a tainted room. It may not be irrelevant to remark, in connection with this fact, that the great proportion of cases of erethema anatomicum have arisen from the examination of persons recently dead of inflammation of the bowels.

In the investigation of this disease, it is not sufficient for the student simply to know that it consists in fever with peritoneal inflammation. Fevers as well as inflammations, may differ, both in degree and in type, and it becomes important to ascertain whether this difference may not be so great as to require a modified, or even an opposite mode of treatment. For the purpose of deciding this, the author proposes to revise the history of the successive epidemics which have appeared for the last half century, and the mode of treatment which is represented to have controlled them most successfully.

A form of puerperal fever is described by Dr. Butler, of Derby, as prevalent between 1765 and 1775, in which bleeding was injurious, and which was cured by purges of Rhubarb, taken every day until the stools became natural. Dr. John Clarke describes a fever prevalent in 1787, which was almost universally fatal. The disease recorded by Dr. Clarke, was accompanied by the symptoms and post mortem appearances by which we recognize puerperal fever at this day; that which Dr. Butler describes, was a chronic disease, often of five or six weeks' duration, and of a remittent character, probably autumnal fever.

Dr. William Hunter found the disease so fatal, that, in his lectures, he uses the following language: "Of those attacked by this disease, treat them in what manner you will, at least three out of four will die. We tried various methods, (bleeding, refrigerants, stimulants, &c), but every thing failed." Richter on the other hand, professor of medicine and surgery at Gottingen, an author of equal repute, remarks, "I have often seen the Child-bed fever, and always treated it successfully— The means of preventing the disease, or if it has already begun, for curing it, are timely evacuations by purgatives."

The detail of symptoms, by these two authors, evidently prove that they refer to the same disease. It is therefore difficult to account for the difference in its fatality unless we suppose that the latter had never seen it epidemic, and that the sporadic cases were singularly mild.

Dr. Lowder, a physician in high repute in London, in the latter part of the last century, describes a very aggravated form of the disease in which the patient died in from forty-eight hours to a week from the time of attack. He considered the inflammation erysipelatous, and the fever typhoid. All the patients, he asserts, who were bled died. When the fever was typhoid, he gave bark—in some instances to the amount of a gallon of the decoction daily.

Dr. Denman bled largely, gave an emetic followed by saline purgatives. Dr. Gordon relied principally on large bleedings used at the very commencement without regard to the strength of the pulse, one active purgative followed by daily saline purgatives, and an opiate at night.

In 1782, M. Doulcet, one of the physicians to the Hotel Dieu, acquired so much reputation by his mode of treating this disease, that the Royal Medical Society of Paris were ordered by the government to make a report upon the subject. This treatment consisted in giving an emetic of ipecacuanha, and promoting its operation upon the bowels by Kermes mineral given in combination with olive oil. The success of this treatment depended very much upon its being used at the moment of attack, but when given under these circumstances, it is represented as being invariably successful. It was tried in England with various results. Some practitioners giving attestations to its efficacy more or less qualified—others asserting that it was injurious. The practice however has fallen into disuse, and there is now a strong prejudice against it, rather, perhaps, from a priori reasoning, than from experience of its injurious effects.

A practice somewhat analogous was pursued by Dr. Boer, of Vienna, physician to the lying-in hospital in that city. His treatment consisted in the administration of a secret preparation of antimony, to which he attributed peculiar efficacy, so administered as to produce powerful perspiration. The secret of the preparation he never revealed.

A very valuable work has been written upon puerperal fever by Dr. Armstrong, Dr. Armstrong described this disease, as he has done the other forms of febrile disease, which he has noticed in his works, under the heads of inflammatory and congestive—the one accompanied with strongly marked symptoms of inflammation—the other with those symptoms of depression which arise from congestion. Little complaint of pain was made in the last form of the disease, except upon pressure, and occasionally not even then, although dissections in such cases, revealed traces of inflammation both in the brain and abdomen. The practice of Dr. A. is well known to have consisted in early, copious, and, if necessary, repeated bleeding, followed by active purging, and eventually by calomel and opium.

About the same time, Mr. Hey, of Leeds, published an account of the epidemic which he had treated in a similar manner with like results.

After this retrospect of the mode of treatment prescribed, at different times, by different physicians, Dr. G. proposes the following important question; viz. "whether the puerperal fever, accompanied by an affection of the peritoneum and often epidemic, does not assume different types in different seasons, being sometimes acutely inflammatory and requiring early and active depletion, "at others characterized by debility or what has been called *action without power*, in which depletion however early and actively employed, is useless and pernicious."

Dr. G. proposes two methods of solving this question. 1. A comparison of the records of past, with our experience of recent epidemics, in order to see whether the coincidence of their symptoms warrants the conclusion that they are curable by similar modes of treatment. 2. The result of the same remedies upon different epidemics. He thinks that the former of these sources of information affords only conjectural proof, and that Dr. Armstrong and the profession generally have erred in permitting the experience of a single epidemic to lead them to form too positive conclusions respecting the uniformity of the symptoms in puerperal fever, and inferring, in consequence, that a similar practice would be equally applicable to all succeeding epidemics. As the subject is one of of practical importance, we shall give his reasoning on it.

"In the leading circumstances of the disease there is certainly great uniformity; it almost always commences a few days after delivery, is marked by pain and tenderness of the belly, and a rapid pulse; if not cured, terminates fatally within a week, and, after death, commonly leaves the depositions and effusions of inflammation. Thus far it is very uniform, but no further. To say nothing of its causes, there are at least three things requisite to form the history of a disease:—1st. Its symptoms. 2d. The effects produced by remedies. 3d. The morbid appearances discovered after death. In the history of puerperal fever there is, even in the first and third of these particulars, considerable difference: this is apparent even in the experience of Dr. Armstrong himself, who describes the local inflammatory symptoms as being sometimes very distinct, sometimes very indistinct, and sometimes absent altogether, the patients not only complaining of no pain in the abdomen, but bearing pressure without the slightest shrinking. Compare, too, the symptoms of the disease as described by Dr. Gordon, of Aberdeen, and those described by Dr. Clark, of London. In the appearance discovered after death, there is also this great difference in different epidemics, that sometimes there is the effusion of inflammation, with extensive redness of the peritoneum, at other times the peritoneum is quite pale; in the sequel it will appear that there is a still greater difference in the appearance after death. But if from the symptoms and morbid appearances we pass to the second particular in the history of the disease, namely, the effects of remedies, (which form not only an essential, but the most important part of this history, for the two others are of no value but as they throw light on this,) there is perhaps no disease of which the histories have been so opposite. Ritcher could almost always cure it. Dr. Wm. Hunter and Dr. Clark could scarcely ever cure it. In Dr. Lowder's time, it was observed that every woman who was blooded died. In Dr. Armstrong's time it was observed that every woman who was not blooded died.

Throughout the whole chapter on the pathology of the disease, Dr. Armstrong writes as if he thought that the symptoms during life, and the appearances discovered after death, were infallible guides to the nature and the treatment of the disease. Thus he remarks, that if a practitioner were to see a woman soon after delivery suffering pain in the abdomen, a quick pulse, and the other signs of fever, and after death were to find no other morbid appearance than extensive traces of inflammation in the abdomen, he would at once conclude that the disease was active inflammation, and would in future treat it as such; and, alluding to those writers who have considered the low epidemic fever of child-bed as a different disease from peritoneal inflammation, he says, 'it becomes a matter of great practical consequence whether symptoms and dissections justify such a distinction.' It appears to me that symptoms and dissections cannot settle the question, and that Dr. Armstrong lays more stress on the argument than it will bear. Supposing many cases of a disease, which bore in general a striking resemblance to one another in the symptoms and the appearance on dissection; this would naturally suggest as a strong probability that they would all be affected in the same way by the same remedies: but suppose that on applying these remedies to all these cases, with the same activity, at the same stage of the disease, and as far as can be made out, under the same circumstances, they produced different effects on different cases, some being relieved and recovering, others being made worse and dying; this would be more conclusive evidence of a difference between the cases, than the symptoms and morbid appearances were of their identity. The effects of remedies on a disease, if accurately observed, form the most important part of its history; they are like chemical tests, frequently detecting important differences in objects which previously appeared exactly similar; some as soon as mercury affects the mouth, begin to mend, and rapidly recover; in others, the ulcers begin to spread; and so imperfect are the appearances as guides, that I have known the first surgeons in the profession giving opposite opinions about the same case, and a nose lost from taking the opinion of the majority. The local pains and constitutional disturbance which occur in feeble and bloodless persons, and which are irritated by bleeding and other evacuants, strikingly resembling the local pains and constitutional disturbance which occur in vigorous and plethoric persons, and which the lancet and other evacuants relieve and ultimately cure; yet how many years is it before the young practitioner learns that there are cases apparently so similar, yet really so different, and how to distinguish them,—and how many practitioners are there who never learn it at all! Symptoms and dissections can never do more than suggest probabilities about the nature of a disease and the effects of a remedy on it. A trial of the remedies themselves is the only conclusive proof. Sydenham was so aware of this that he says, 'Epidemic diseases may seem alike to the unwary, because in some sort they do agree to outward appearance;' adding this confession, 'when a new species of fever arose, I was doubtful how to proceed, and notwithstanding the utmost caution, could scarcely preserve over one or two of the first patients from danger,' so far from infallible were symptoms as guides."

It is scarcely necessary to remark upon this subject, that all experience confirms the wisdom of Sydenham's remark. Notwithstanding the proneness of medical men to form general conclusions from particular epidemics, nothing is more certain than that independent of the modification of disease produced by local circumstances, and by peculiarity of temperament and mode of living, there is an atmospheric influence upon diseases, rendering them at one time highly inflammatory, and at another, producing a degree of debility that requires the utmost caution in the use of depleting remedies.

For instance, the yellow fever, on its appearance in the different seaports of the United States, in '93, was every where of an inflammatory character, and this condition, with some modifications, prevailed in its successive attacks for many years.

On the contrary, the typhoid epidemic which commenced in 1812, and for many successive years prevailed throughout the union, was *almost* universally of an opposite character. The character of the epidemics we have since had, has varied, sometimes requiring, at other times contraindicating the use of the lancet. The small-pox, the measles, and other inflammatory diseases are modified by this atmospheric influence, and we see no reason why peritoneal inflammation should not obey the general law. But we proceed to give Dr. G. an opportunity of verifying his logic by his own experience. Preparatory to this, we take the liberty of quoting his admirable description of the disease.

"The cases which were so numerous in those unhealthy seasons had the common symptoms and course of puerperal fever. They began a few days after delivery; the leading symptoms were diffused pain and tenderness, with some swelling of the abdomen, a quick pulse, which was generally at first full and vibrating. Sometimes it was small, but still it was hard and incompressible; the skin was hot, though not so hot as in other fevers; the tongue was white and moist, the milk was suppressed. As the disease advanced, the belly became less painful, but more swelled, and the breathing short; towards the end, the pulse was very frequent and tremulous, and the skin covered with a clammy sweat; even in this state the tongue continued moist and the mind clear, and death took place generally about the fifth day. On opening the abdomen, which was often as large as before delivery, the intestines were found distended with air, the peritoneum was red in various parts, its surface was covered with a coat of lymph, the intestines adhered to one another, and the omentum to the intestines; coagulable lymph was deposited on various surfaces, especially in the depressions between the convolutions of the bowels and on the omentum, on both which parts it often lay in large masses; the cavity of the peritoneum contained several pints of a turbid fluid, apparently serum mixed with lymph. In the uterus the morbid appearances were generally confined to its peritoneal covering, which was coated with lymph, on removing which the membrane itself was found unnaturally red; but in some cases the disease had penetrated deeper into the uterus, the substance of which was sometimes infiltrated with pus, and sometimes contained small abscesses about the size of a nut; the inner surface of the uterus especially at the fundus, often appeared black and ragged, as if gangrenous. The enlargement of the abdomen depended entirely on air in the intestines; when there was no air there was no enlargement, even though the peritoneum contained several pints of fluid. The first time I notived this was in the body of a young woman who had died with all the symptoms of puerperal fever excepting the tumid belly. When the body was lifted from the bed on to the board on which it was to be opened, the belly instead of being tumid was sunk and hollow, and we began to think that her case had been mistaken; but on opening the abdomen we found several pints of turbid fluid.

The disease generally began very suddenly. After being quite well, feeling no sense of illness, or at least making no complaint, the patient was seized at once with chilliness, or shivering, and pain in the belly, and the pulse rose to 120 or 130; but sometimes the attack was more gradual. For many hours, or even for a day or two, there were pain and tenderness in one part of the abdomen, then in another, with long intervals, in which there was no pain anywhere; and during all this time the pulse would remain quiet, or not quicker than 80 or 90. In short the disease would have an incipient stage, but this was not a common occurrence."

The following is the mode of treatment which the author, after unsuccessfully trying other methods of cure, was led to adopt.

"I now found that, provided I saw the patient within a few hours of the attack, I could generally arrest the disease. The mode of treatment was as follows. A vein was opened in the arm, with a wide orifice, so that the blood flowed in a full stream, and it was allowed to flow till the patient felt faint; the arm was then tied up, and her head was raised so as to encourage the faintness for many minutes. As soon as the faintness had subsided, she took from ten to twenty grains of calomel in a tea-spoonful of arrow-root, and afterwards half an ounce of sulphate of magnesia dissolved in beef-tea or thin gruel, every other hour, until several copious evacuations were procured from the bowels; when the patient had thoroughly recovered from her faintness, from ten to twenty leeches were applied to the painful and tender parts of the abdomen; when the leeches had fallen off, a bag long and broad enough to cover the whole abdomen, was stuffed with hot poultice which was spread so as to form a cushion nearly an inch thick; this was laid hot over the whole abdomen, and renewed so often as to keep up heat and moisture. If the patient complained of the weight of the poultice, the bag was stuffed with scalded bran. We found this application of infinite value, not only as a means of encouraging the bleeding of the leech bites, but also as a perpetual fomentation."

Dr. G. insists strongly on the importance of keeping in view the different object of local and general blood-letting. The former relieves the general inflammatory diathesis—the latter relieves local congestion. Leeches cannot fulfil the indications which require the lancet, and would be useless so long as the activity of the circulation is kept up by the undiminished action of the heart. When this has been subdued, local depletion will relieve the pain and vascular engorgement which may continue after general depletion has been carried as far as prudence will justify.

After waiting the combined effects of these remedies, Dr. G. determined whether a second bleeding was to be resorted to or not; but it is to be borne in mind that the active treatment which is to determine the fate of the patient should be completed during the first day, for so rapid is the tendency to peritoneal effusion, that the later application of remedies is attended with very diminished chances of success.

After full depletive measures had been resorted to, sufficient tenderness sometimes remained to oceasion anxiety about the fate of the patient, which were promptly relieved by a full opiate, and a warm poultice to the abdomon.

Dr. G. also remarks that the large and repeated doses of calomel which he was in the habit of giving, frequently produced soreness of the gums, and that he never saw a case terminate fatally after this event took place, although the symptoms were sometimes of the most alarming character.

The author illustrates his mode of treatment by a number of cases which we are compelled to pass over. He then remarks, that in common with the rest of the profession, he entertained the opinion, for a number of years, that the group of symptoms which indicate puerperal fever. uniformly pointed out a similar disease, to be combatted, under all circumstances, by a practice conducted upon similar principles. But a number of cases having fallen under his observation which terminated fatally under the usual depleting remedies, and in which no traces of inflammation were found on dissection, and of others which were suc-fully treated by opiates and fomentations, he was led to adopt different views. We shall leave him to express his own conclusions.

"These cases opened to me a new view of the subject; they taught me that lying-in women might have permanent pain and tenderness of the abdomen, with a rapid pulse, and independent of acute inflammation of the peritoneum, or any other part; that these symptoms may depend on a state which blood-letting does not relieve, and which, if this remedy is carried as far as it requires to be carried in peritonitis, may terminate fatally; and that the most effectual remedies are opiates and fomentations. Most of the patients who were the subjects of these attacks were women who in their ordinary health were delicate and sensitive. The attack sometimes seemed to originate in violent after-pains, gradually passing into permanent pain and tenderness, resembling inflammation, or in the painful operation of an active purgative; but it could sometimes be traced to no satisfactory cause; the patient had a common labor, and had experienced no unusual cause of debility or irritation. The pulse in all of these cases, although quick, was soft and feeble: this together with the previous constitution of the patient were my chief guides; when I could trace it to any irritating cause, such as a griping purge, and when blood had, been already drawn without relief, and without being buffed, I saw my way still clearer. When I doubted, I applied leeches to the abdomen.

The cases which I have related, and others similar to them, were speedily and completely relieved by the remedies which I have mentioned. There seemed to be nothing dangerous in this form of disease, provided the nature of it was not mistaken, and improper remedies not used; yet it so strikingly resembled peritoneal inflammation, that it was invariably taken for it by the practitioners who witnessed it, all of whom possessed at least that average quantity of sense and knowledge on which the public must extensively depend. These cases though sufficiently numerous to attract my notice, and produce a strong impression on my mind, were nevertheless only occasional occurrences. I had never seen them in numbers at the same time; they occurred at comparatively long intervals, and at times when there was no prevalent disease among lying-in women; they resembled sporadic diseases both in the rarity of their occurrence, and in the facility with which they were cured. At the hospital I saw two or three cases, in which after the ordinary symptoms of puerperal fever had terminated fatally, and the body was opened, the usual morbid appearances were not found; nothing appeared but a moderate quantity of fluid slightly stained with blood.

The author remarks that these and many similar cases have convinced him that there is a form of peritoneal fever which, although it has the ordinary symptoms, is very different from the peritoneal fevers which prevailed between 1810 and 1820—different in its duration which is shorter; in the way in which it is affected by blood letting; and lastly in the morbid appearances discovered after death. It may, like the acute inflammatory form, occur sporadically, when it is easily cured by opiates, fomentations, aperients, assisted sometimes by leeches, or it may be epidemic, and consequently more malignant.

The most remarkable circumstance which the experience of the last few years had taught us about peritoneal fever is, that they may occur in their most malignant and fatal form, and yet leave few or no vestiges in the peritoneum after death. The state of this membrane, indicated by pain and tenderness of the abdomen, with a rapid pulse, appears to be not one uniform state, but one which varies so much in different cases, that a scale might be formed of its several varieties; this scale would begin with little more than a nervous affection often removable by soothing remedies, and when terminating fatally, leaving no morbid appearances discoverable after death. Next above this, a state in which this nervous affection is combined with some degree of congestion, indicated in the cases which recover, by the relief afforded by leeches, and in the cases which die, by slight redness in parts of the peritoneum, and a slight effusion of serum, sometimes colorless, sometimes stained with blood. Above this might be placed those cases in which there are, in the peritoneum, the effusions of inflammation without its redness, namely, a pale peritoneum and no adhesions, lymph like a thin layer of soft custard, and a copious effusion of serum rendered turbid by soft lymph. Lastly, the vestiges of acute inflammation of the peritoneum, namely, redness of this membrane, adhesion of its contiguous surfaces, a copious effusion of serum, and large masses of lymph.

Dr. G., in concluding the paper, thinks himself justifiable, from the history of the cases he has published, in deciding that the disease, under different circumstances, possesses different characters, and requires a diversified treatment. His opinion could not be better expressed than in the following quotation from Sydenham.

Sydenham, who, in speaking of epidemic diseases, tells us that their difference is apparent not only from their peculiar symptoms, but also from the different methods of cure they respectively require; that though they seem to agree in their external face, and certain symptoms in common, they are in reality of very different and dissimilar natures; that the same method which cures in the middle of the year may possibly prove destructive at the conclusion of it; and that, when a new species arose, he was doubtful how to proceed, and, notwithstanding the utmost caution, could scarce ever preserve one or two of his first patients from danger.' There are no diseases to which these remarks are more applicable than the peritoneal fevers of lying-in women.

For combatting the different forms under which the disease presented itself, Dr. G. recommends five as possessing the greatest body of evidence in their favor.

1. *Bleeding and purging.*

Thus, when the prevailing diseases are inflammatory, and are best cured by depletion, when the pulse at the beginning of the disease is vibrating, hard, or, although small, is incompressible, when the skin is not only hot, but dry, and when, after the treatment of a few cases, it appears that those have recovered best in which bleeding and purging have been used early and fairly, there is every reason to believe that we have lighted on an inflammatory epidemic, and that depletion is the means on which we must depend for the cure.

2. *Emetics of Ipecacuanha.*

The conditions under which they ought to be given are these:—1. they are to be used at the instant of attack; 2. they are to be repeated every day till the symptoms are subdued; 3. in the intervals between the operation and the emetic, a potion is to be given, composed of oil of almonds, syrup of marshmallows, and Kermes' mineral; 4. the diet is to consist of nothing but linseed tea, or something equally bland and unheating.

3. *Calomel.* The efficacy of mercury in inflammations of the eye, the liver, and the pericardium, and the inadequacy of blood letting to overcome inflammation in these organs without its assistance, give it a claim to a trial, in peritoneal inflammation. Dr. G. has never relied upon it. He has often given it, however, and has never seen a patient die when salivation was produced; although in some cases the disease has been fatal before salivation could be produced. It is now, we believe, generally admitted, that the constitutional effects of mercury can seldom be relied upon where blood letting is clearly indicated. During the use of calomel, opium is generally necessary, especially if there be severe pain, profuse purging, or a sinking of the general circulation. When the gums are sore, or the disease has yielded, the calomel should not be suddenly withdrawn, but continued for some time in smaller doses and at longer intervals.

4. *Opiates internally, and hot Poultices to the abdomen.*

There is a class of cases attended by pain and tenderness of the abdomen, with a rapid pulse, which does not require bleeding, which does not bear it to the extent to which it is necessary in the inflammatory peritoneal fevers, and which is speedily and effectually cured by opiates internally, with hot poultices to the belly, aperients, and sometimes leeches. There is reason to believe that this form of the disease is present when the patient in her ordinary health is delicate and nervous—when the pain and tenderness have followed any irritating cause, such as severe after-pains, or a griping purge—when the pulse, although quick, is perfectly soft, and even weak, and this opinion is strengthened if blood has been drawn without relief, and without the signs of inflammation on its surface. The best way of treating these cases is to wash out the large bowels by a very large glyster, to give ten grains of compound powder of ipecacuanha every three hours, till the pain is gone, to keep the abdomen constantly covered with a warm linseed meal poultice, and after the pain has ceased, if the abdomen continues sore and the pulse quick, to apply leeches, and give a mild purge. When I doubt the nature of the case, I apply leeches at the beginning."

5. *Oil of Turpentine.* Although Dr. G. has not been successful with this remedy in his own practice, he thinks we are authorized to conclude from the testimony of competent witnesses, that there is a class, or, perhaps, a stage of these fevers in which it is highly efficacious. He does not however decide upon the circumstances in which it is proper, nor the manner in which it is to be given.

Chapter II. *On Disorders of the Mind in Lying-in Women.*

Dr. G. remarks, that during the whole period of pregnancy and nursing, females are liable to disorders of the mind, but there are two periods when they are peculiarly liable to occur—"soon after delivery, when the body is sustaining the effects of labor"—or, "several months afterwards, when the body is sustaining the effects of nursing." When it occurs as the consequence of nursing, it generally appears in the form of melancholia. Dr. G. does not give a general description of its symptoms, but refers his readers to a number of cases which he has recorded, and which are indeed truly valuable. For these we must refer our readers to the work itself, having room to give only a general idea of the causes of the disease, its character, and the most suitable remedies.

Of the causes, one of the most important is undoubtedly *constitutional predisposition.* Dr. G. remarks.

> Of the causes which I have seen, a large proportion have occurred in patients in whose families disordered mind had already appeared. The patients too were of susceptible dispositions, nervous, remarkable for an unusual degree of that peculiarity of nerve and of mind, which distinguishes the female from the male constitution.

2. *Disordered condition of the Digestive Organs.* In one of Dr. G's patients, three successive attacks were connected with jaundice, and in a fourth pregnancy, the jaundice having been relieved before her confinement by suitable remedies, she escaped the disease. In some other cases the disease was manifestly connected with this condition, and promptly relieved by correcting it.

3. The disease has been attributed to *suppression of the milk*, in consequence of weaning; but Dr. G. states he has never known it to occur in those females who never nurse their children, and suppress their milk without ever having their breasts drawn; and where disease has occurred at a later period, the patient has been directed to wean the child, because she had not strength to enable her to nurse it. The mind has sympathized with the gradual impairment of the health, and to depression of spirit, listlessness, and loss of memory, has succeeded derangement.

4. It has been also attributed to *suppression of the lochia*, and although we might admit with Dr. G. that it would be absurd to attempt the cure of the disease by restoring this discharge, we cannot, especially, if with the author, we admit uterine derangement to be the efficient cause of the disease, treat the idea as entirely absurd.

5. The opinion of Dr. G. is, that the cause of the disease is to be sought for in *that peculiar state of the sexual system which occurs after delivery.*

The sexual system in women is a set of organs which are in action only during half the natural life of the individual, and even during this half they are in action only at intervals. During these intervals of action they diffuse an unusual excitement throughout the nervous system; witness the hysteric effects of puberty, the nervous susceptibility which occurs during every menstrual period, the nervous affections of breeding and the nervous susceptibility of lying-in women. I do not mean that these appearances are to be observed in every instance of puberty, menstruation, pregnancy, and child-bed, but that they occur sufficiently often to show that these states are liable to produce these conditions of the nervous system.

The prognosis in this disease is for the most part favorable. Some however die of this disease, and the prognosis therefore is to be guarded. Dr. Baillie is said to have remarked in relation to a patient who died soon after, "that the question was not whether she was to get well, but when she was to get well." The opinion of Dr. G., supported by that of Dr. Hunter, is that there are two forms of puerperal mania, the one attended by fever, or at least by a very rapid pulse, the other accompanied by a very moderate disturbance of the circulation; that the latter cases which are by far the most numerous recover; that the former generally die."

As to the duration of the disease, Dr. G. remarks that mania is a less durable disease than melancholia—more dangerous to life, but less dangerous to reason. Of the many cases about which Dr. G. had been consulted, only two remained permanently deranged, and one of these had been so before her marriage. He has known but one case in which the disease returned a second time.

*Treatment.* 1. The attendants upon the patient should be such as will effectually controul her by mild but decided conduct—a task that can seldom be well executed by her own servants or relatives.

2. As there is often a propensity to suicide, every means and opportunity for self injury should be carefully guarded against. Experience is in favor of exclusion from society, especially from that of relations. "*The husband should never be left alone with his deranged wife for obvious reasons. I have known more than once a neglect of this rule produce consequences which, left in the minds*

*of those concerned, a never-ending regret. On this subject, a candid appeal should be made to the sense and feelings of the husband.*" Cases may occur in which some interruption to this solitary life may be advisable.

When the disease has lasted long, when the patient expresses a strong wish to see some near friend, when she entertains illusions which the sight of some one may efface, the admission of such person is worth a trial. I shall be told, that when patients are mending, or have recovered, the most common cause of relapse is too early an introduction to friends, and too early a return home. When the patient is recovering, or has recovered, I do not recommend these measures.—It is when the patient has not recovered, and is not recovering, that I advise them to be tried; when month after month passes without any amendment, and her mental delusions assume a shape accessible to moral impressions, then it is that I would advise an interview with a friend.

The diet should generally be as nutritious and cordial as the impaired condition of the digestive organs will admit of the patient's using.

The medicinal agents which have been employed are

1. *Blood letting* general or local. Dr. G. thinks this remedy is almost always injurious. If there are marks of inflamation or congestion of the brain, local blood letting may be used, although with very great caution. Of the cases with a quick pulse which Dr. G. has seen, those only have recovered in which this remedy was not resorted to.

2. *Emetics and purgatives*, to evacuate the primæ viæ and amend the secretions which flow into them. The influence of emetics upon other mental disorders, would recommend their employment in this disease, especially if the symptoms of gastric derangement are present. Ipecacuanha is preferable to the preparations of antimony. It should be given in a full dose and its operation promoted by a free use of warm water. Where there is any reason to suspect bilious derangement or irritation of the bilious secretions, such purgatives will be selected as will operate effectually and with as little expense to the powers of the stomach or of the general system as possible—blue mass, jalap, aloetic preparations, &c.

3. *Narcotics*, are in the opinion of the author, the most valuable remedies in this disease. As soon as the flush of the cheek and other slight febrile symptoms which accompany this disease are relieved, such narcotics as agree best with the patient should be given systematically until the patient is brought under their influence. When this effect is obtained it should be kept up by diminished doses repeated at proper intervals, and occasional full doses if necessary. When opiates have effected their object they should be withdrawn gradually by diminishing the dose and lengthening the interval, carefully watching their effects, prepared to renew them if any injurious effects result from this suspension.

4. If the disease becomes protractod, the strength is to be sustained by the *mineral acids*, *bitters*, or under some circumstances, bark.

Chapter III contains many interesting remarks, with some valuable eases in illustration, *upon the mode of distinguishing Pregnancy from the diseases which resemble it*,

In illustration of the fallibility of the ordinary signs of pregnancy, the author has the following remarks.

1. Many women assert that they have menstruated regularly during the early months of pregnancy: whether this is really menstruation, or a periodical hæmorrhage from partial separation of the ovum, is not the question, but whether, during the first months of pregnancy, there may not occur a monthly discharge of blood which in period and duration so far resemble menstruation, that the patient is unable to distinguish it; and about this there can be no doubt. As to sickness, there is an infinite variety in the degree in which it occurs in pregnancy; some persons are sick day and night, during all the nine months; others never feel the slightest nausea from the moment of conception to that of delivery. In thin women the enlargement of the breasts is often very slight; and in fat women the breasts form so small a proportion of the bosom, that any enlargement of the former is scarcely perceived. In very fair women with light hair and eyes, the discolouration of the areola is often so slight, that it is difficult to perceive it; and in brunettes who have already borne children, the areola remains dark ever afterwards, so that this ceases to be a guide in all subsequent pregnancies. The enlargement of the abdomen from the third month to delivery, is in all cases present and progressive whilst the fœtus is alive; but it may die, and yet be retained till the ninth month, in which case the enlargement will not be progressive. The same may be said of the movement of the fœtus; it will not move if not alive, and there are cases, though rare, in which it has not moved during the whole of pregnancy, although it has been born alive and vigorous; of this I have known one instance, and read of others. Thus a woman may be pregnant though she seems to herself to continue to menstruate, has no sickness, or enlargement about the breasts, or darkness of the areola, or progressive enlargement of the abdomen, or perceptible movement of the fœtus. Such a complete assemblage of omissions however is not likely to meet in the same case.

2. A woman may have apparently all the symptoms of pregnancy, and yet not be pregnant: menstruation may be stopped by other causes; when it ceases suddenly in a woman of healthy constitution, who had previously menstruated with perfect regularity, it is a strong symptom, but there are women of feeble constitutions who, without being pregnant, frequently pass months without menstruating; in such a person the omission of menstruation proves nothing. Sickness may be produced by other causes besides pregnancy; and when it arises from a weakness of stomach, the morning is the time when it is most distressing. The bosom may enlarge because the patient is growing plump. Some have laid great stress on the darkness of the areola, thinking that no other state besides pregnancy is capable of producing it. I saw two young and newly-married women within two days, who had made preparations for lying-in, and who were not pregnant. In both, the areola was dark, though (if their history is to be trusted) they never had children. I believe, however, that darkness of the areola rarely depends on other causes, and that when it exists it may generally be looked upon as a sign either that the patient is pregnant, or has been so formerly. The abdomen often enlarges from flatulence, dropsy, and other diseases so as to equal or exceed the bulk of pregnancy. As to the movements of the child, it is very important to distinguish between these movements as felt internally by the patient, and as felt externally by a hand applied to the surface of the abdomen; the latter, if really felt, is an infallible sign of pregnancy, but the former are often felt when there is no child. Thus a woman may cease to menstruate, have sickness, enlargement of the bosom, and darkness of the areola, a progressive enlargement of the abdomen, and sensations which resemble those produced by the movements of the child, without being pregnant.

The occasional uncertainty of all these signs, renders something additional requisite. Whenever therefore a positive decision upon the subject becomes necessary, an *examination by touch* should be resorted to.

In this examination two cases are to be decided. 1st. whether the enlargement of the abdomen depends upon enlargement of the uterus, and if so, 2nd, whether the enlarged uterus contains a foetus. The examination may be made, either *external-*

mour distending the abdomen.

We are next to attend to the appearance of the umbilicus. When the uterine tumor rises to the level of the umbilicus, or a little above it, the depression at the umbilicus is gradually elevated, and by the seventh or eighth month is entirely removed. The elevation of the umbilicus may be produced by any other firm tumor distending the abdomen, but its absence at a late period of imaginary pregnancy, proves that the distension does not proceed from a foetus.

The motion of the child which may almost always be produced by suddenly laying the cold hand upon the belly, is among the most infallible signs.

On making the internal examination, we attend first to the neck of the uterus. This in the unimpregnated uterus is a firm fleshy nipple, projecting into the vagina two thirds of an inch. The obliteration commences about the fifth month, later in first pregnancies, the neck becoming broader, softer and shorter. The neck is not entirely obliterated until a week or two before parturition.

The enlargement of the uterus is the next point of enquiry. This information will be more certainly obtained by pressing at the same time upon the tumor externally.

The child in the uterus is surrounded by the liquor amnii, with the head, when the woman is in the erect position, resting upon the os uteri. By suddenly pushing the distended uterus, the head of the child recedes from the point of the finger, and again, a moment after, is felt falling upon it. This is a proof of pregnancy not less certain than the motion of the child, and has this advantage over the latter that it can be felt whether the child be dead or alive.

CHAPTER IV.—*On Polypi of the Uterus.*—Dr. G. thinks that this disease is of far more frequent occurrence than is generally supposed, and that it is very frequently mistaken for hemorrhage from the uterus.

"A polypus of the uterus, when discovered, is a tumour in the vagina attached to some part of the uterus. It is round, smooth, firm, and insensible; it is quite unattached to the vagina, so that the finger can be pressed round between the walls of the vagina and the surface of the tumour; but if traced higher up, it is found to terminate in a narrower part of stalk. This stalk is differently attached in different cases; in some it passes through the orifice of the uterus into its cavity, and is attached to the fundus of this organ; in others, it passes into the cavity of the neck, to one side of which it is attached; in others it does not enter the orifice, but is attached to one portion of its edge or lip; hence a distinction of polypus of the fundus, polypus of the neck, and polypus of the orifice. This distinction must not be lost sight of, for it is of practical consequence. In ascertaining the nature of the tumour for the purpose of determining the propriety of removing it by an operation, the mode of its attachment is one of our chief guides; and in this respect what is true of polypus of the fundus, is not so of polypus of the neck or lip.

In polypus of the fundus the stalk is completely encircled by the neck of the uterus, and if the finger can be introduced into the orifice, it passes easily round between the stalk of the polypus and the encircling neck.

In polypus of the neck the finger cannot be passed quite round the stalk; it may be passed partly round it, but it is stopped when it comes to that part where it is attached to the neck, the stalk is only *semi*-circled by the neck.

In polypus of the edge of the orifice or lip, the stalk does not enter the orifice, but grows from the edge of it; it feels as if a portion of the lip was first prolonged into the stalk, and then enlarged into the body of the polypus. It is important to remember that there is a polypus, the stalk of which is not encircled by the orifice of the uterus; if it grows from the orifice it cannot be encircled by it.

When a polypus grows within the uterus, it dilates its cavity, neck, and orifice, as in pregnancy. Instead of the orifice of the projecting part of the neck forming a narrow chink in a firm thick nipple, it is a round space with thin edges, as in somewhat advanced pregnancy. In polypus of the neck and that of the lip, the projecting part of the uterus preserves more of its ordinary form and consistence."

The tumors most likely to be mistaken for a polypus are: 1, the prolapsed uterus; 2, the inverted uterus; 3, malignant excrescences from the uterus. The sensibility of the tumour, the well defined form of the uterus, the obstruction to passing the finger, from the fold of the vagina, and the relief produced by pressing up the tumor, would, we presume, enable the most hasty observer to distinguish the former of these from polypus.

The diagnosis between inverted uterus and polypus is more perplexing,——the most distinguishing marks are, the circumstances under which the former occurs, almost always immediately after parturition, and the sensibility of the tumor.

A more frequent subject of doubt is whether a tumor projecting from the uterus into the vagina is a common polypus admitting of a cure, or a malignant excrescence. The diagnosis is very difficult Dr. G. recommends that in all cases, where there is a stalk admitting of the application of a ligature without danger of including the uterus, the experiment should be tried and that it will very generally succeed, altho' the general character of the tumor may indicate a disease of much more serious character.

If polypus of the uterus is overlooked or neglected, the patient generally dies of hemorrhage. Dr. G. therefore recommends that whenever a flow of blood from the uterus proves obstinate an examination by touch should be instituted. If polypus exists, its removal should be immediately attempted either by the knife or by ligature.—He prefers the latter as more safe and successful.

Unpleasant accidents have sometimes followed the application of the ligature. Dr. Denman lost a patient under these circumstances, and after death the ligature was found applied to the inverted uterus. The same accident occurred to Dr. Hunter, and, what rendered the case more remarkable, in a female who had never been pregnant. Dr. G. and several other practitioners have been similarly unfortunate.

By observing the following rules, Dr. G. thinks the accident may be avoided: 1 Pass the ligature as low upon the neck of the tumor as possible, taking care to include the body of the tumor. When the tumor is separated the stalk will die and fall away. 2, If the polypus arise from the cervix uteri, the os uteri if it can be felt will be the best guide; if it cannot be distinguished, sufficient allowance must be made for the usual length of the cervix. 3, To attend to the sensations of the patient when the ligature is tightened. As soon as the ligature is tightened the hemorrhage ceases, & after a time a fetid discharge takes place from the vagina which continues until the separation is effected. This occurs sooner or later according to the size of the neck, and the frequency with which the ligature is tightened—from two to eight or ten days. When the polypus is of small size it comes away of itself—if larger it is expelled by the contractive power of the uterus, or manual interference may be necessary.

The size of the stalk of the tumor, Dr. G. thinks, is no further an objeetion to the operation than that it renders it more tedious. It is also his opinion that the tumor is generally single, and consequently, that when one is removed the patient may for the most part be considered as cured.

In the conclusion of the chapter the author speaks of some unusual circumstances accompanying polypi :

"In a vast proportion of cases—to speak in numbers, in nine out of ten—to whatever part the stalk grows, frequent hæmorrhages from the uterus form the symptoms of the disease, which attracts the chief attention of the practitioner, and the case is long mistaken for common menorrhagia; but when the tumour grows from the neck or lip of the uterus, it sometimes occasions nothing but an obstinate and profuse leucorrhæa.

Women who have a polypus of the uterus, especially if it grows from the neck or lip of this organ, sometimes become pregnant. Of this I have known two instances. In one the tumour was discovered in the fifth month of pregnancy, and was removed by the ligature. The pregnancy went on to the ninth month, when the patient was safely delivered. In the other case the tumour was not discovered till the commencement of labour, and occasioned the death of the patient a few hours after delivery.

When the polypus grows from the lip of the uterus, and its stalk is very thick, as two or three inches in diameter, the other lip and the orifice of the uterus are quite lost, and cannot be detected by touch.

I have seen the most experienced practitioners in London puzzled to tell what was the nature of the tumour, and what ought to be done for it.

A polypus may have an unusual form, as that of a solid cylinder ; it is generally globular or pyriform, with a stalk like an elastic gum bottle; but I have seen two cases in which it was cylindrical, about the size of half the wrist, and the unusual form occasioned great doubts about the nature of the case and the mode of treatment.

A polypus is sometimes so small, that it seems incredible it should occasion the frequent hæmorrhages which attend it, yet the hæmorrhages cease on the removal of the polypus. I have felt them as small as a filbert without its shell, growing to the neck or lip of the uterus; they were so small, that on being touched they slipped into the orifice of the uterus, and there remained concealed till the finger was withdrawn, and the patient sat up, and they dropped down again into the vagina.

These little polypi are generally too small for the ligature ; and if they cannot be pulled away with the finger, should be twisted off by a pair of surgeon's dressing forceps."

Chapter V. is devoted to the *Irritable uterus*—"a painful and tender state of this organ, neither attended by, nor tending to produce, change in its structure," a species of neuralgia analogous to what Sir A. Cooper has described under the name of irritable tumour of the breast. Mr. Brodie has also described a similar disease of the joints, both occurring chiefly amongst hysterical females.

The following is Dr. G's. description of the disease,

"A patient who is suffering from the irritable uterus complains of pain in the lowest part of the abdomen, along the brim of the plevis, and often also in the loins. The pain is worse when she is up and taking exercise, and less when she is at rest in the horizontal posture; in this respect it resembles that of prolapsus uteri, but there is this difference, that in the latter, if the patient lies down, she soon becomes quite easy; but in the complaint of which I am speaking, the recumbent posture, although it diminishes, does not remove the pain. It is always present in some degree, and severe paroxysms often occur, although the patient has been recumbent for a long time. If the uterus is examined, it is found to be exquisitely tender, the finger can be introduced into the vagina, and pressed against its sides without causing uneasiness, but as soon as it reaches and is pressed against the uterus, it gives exquisite pain.

The pulse is soft, and not much quicker than is natural; but it is easily quickened by the slightest emotion. In a few instances, however, there has been a greater and more permanent excitement of general circulation; the degree in which the health has been reduced has been different in different cases. A patient, who was originally delicate, who has suffered long, and has used much depleting treatment, has been (as might reasonably be expected) the most reduced; she has grown thin, pale, weak and nervous; menstruation often continues regular, but sometimes diminishes, or ceases altogether; the functions of the stomach and bowels are not more interrupted than might be expected from the loss of air and exercise; the appetite is not good, and the bowels require aperients; yet nothing more surely occasions a paroxysm of pain than an active purgative.

The causes to which this disease has been attributed, and after the application of which it has occurred, are generally considerable bodily exertions at times when the uterus is in a susceptible state. In one patient it came on after an enormous walk during a menstrual period; in another, it was occasioned by the patient's going a-shooting with her husband not many days after an abortion; in a third, it came on after standing for several hours many successive nights at concerts and parties; in a fourth, it originated in a journey in a rough carriage over the paved roads of France; in a fifth, it was attributed either to cold or an astringent lotion, by which a profuse lochia was suddenly stopped, followed by intense pain in the uterus; in a sixth it occurred soon after, and apparently in consequence of, matrimony. Although however, the disease followed, and was apparently excited by these several causes of irritation, yet the patients had previously manifested signs of predisposition to it; they were all sensitive in body and mind, many of them had been previously subject to the ordinary form of painful menstruation. The disease seemed to consist in a state of the uterus similar to that of painful menstruation, only permanent instead of occasional."

As to the treatment, the author has nothing satisfactory to offer. The different authors who have treated on neuralgia may be consulted.

Chapter VI. is devoted to a *Peculiar form of the Hemorrhage from the uterus*, described as occurring after such a degree of uterine contraction has taken as is generally supposed to secure the patient against hemorrhage and taking place in the same female in successive labour. Dr. G. recommends the left hand to be passed at once into the womb to the position occupied by the placenta and there to act as a tourniquet while counter pressure is made with the right upon the parietes of the abdomen. When a female is discovered to be liable to this form of hemorrhage, proper attention should be paid to the circulation during pregnancy and parturition. F.

